# Chirality and Magnetocaloricity in GdFeTeO_6_ as Compared to GdGaTeO_6_

**DOI:** 10.3390/ma14205954

**Published:** 2021-10-10

**Authors:** Elena Zvereva, Tatyana Vasilchikova, Maria Evstigneeva, Angelica Tyureva, Vladimir Nalbandyan, João Gonçalves, Paolo Barone, Alessandro Stroppa, Alexander Vasiliev

**Affiliations:** 1 Department of Low Temperature Physics and Superconductivity, Physics Faculty, Lomonosov Moscow State University, 119991 Moscow, Russia; zvereva@mig.phys.msu.ru (E.Z.); t_vasilchikova@yahoo.com (T.V.); 2 Department of Chemistry, Southern Federal University, 344090 Rostov-on-Don, Russia; maevstigneeva@gmail.com (M.E.); tyureva.lika@mail.ru (A.T.); vbn@sfedu.ru (V.N.); 3 Department of Physics and CICECO, University of Aveiro, 3810-193 Aveiro, Portugal; joaonsg@ua.pt; 4 Consiglio Nazionale delle Ricerche, Institute for Superconducting and Innovative Materials and Devices (CNR-SPIN), Area della Ricerca di Tor Vergata, Via del Fosso del Cavaliere 100, I-00133 Rome, Italy; paolo.barone@spin.cnr.it; 5 Consiglio Nazionale delle Ricerche, Institute for Superconducting and Innovative Materials and Devices (CNR-SPIN), c/o Department of Physical and Chemical Sciences, University of L’Aquila, Via Vetoio Coppito, I-67100 L’Aquila, Italy; alessandro.stroppa@spin.cnr.it; 6 Quantum Functional Materials Laboratory, National University of Science and Technology “MISiS”, 119049 Moscow, Russia

**Keywords:** chirality, magnetocaloricity, metaloxide

## Abstract

GdFeTeO_6_ and GdGaTeO_6_ have been prepared and their structures refined by the Rietveld method. Both are superstructures of the rosiaite type (space group $P\bar{3}1c$). Their thermodynamic properties have been investigated by means of magnetization *M* and specific heat *C*_p_ measurements, evidencing the formation of the long-range antiferromagnetic order at *T*_N_ = 2.4 K in the former compound and paramagnetic behavior down to 2 K in the latter compound. Large magnetocaloric effect allows considering GdFeTeO_6_ for the magnetic refrigeration at liquid hydrogen stage. Density functional theory calculations produce estimations of leading Gd–Gd, Gd–Fe and Fe–Fe interactions suggesting unique chiral 120° magnetic structure of Fe^3+^ (*S* = 5/2) moments and Gd^3+^ (*J* = 7/2) moments rotating in opposite directions (clockwise/anticlockwise) within weakly coupled layers of the rosiaite type crystal structure.

## 1. Introduction

Both classical and quantum spin systems of reduced dimensionality host a plethora of exotic magnetic ground states, including spin liquids and peculiar long-range ordered patterns [1,2]. The layered crystal structure of the rosiaite type, AB_2_O_6_, can be considered as an ideal playground to study the low-dimensional and frustrated magnetism. It is organized by diluted triangular *A* and dense honeycomb *B* layers alternating along the trigonal axis *c* [3]. In case of the *A* position being occupied by the divalent transition metal ions (*M* = Mn, Co, Ni), the long-range antiferromagnetic (AFM) order takes place at low temperatures in both *M*As_2_O_6_ arsenates [4] and *M*Sb_2_O_6_ antimonates [5]. Exceptionally, CuSb_2_O_6_ [5] does not order down to 1.5 K, while PdAs_2_O_6_ orders at an unusually high Néel temperature 140 K [6]. The couple of pentavalent cations in the *B* layer can be substituted by a combination of tetravalent and hexavalent cations, as is the case of MnSnTeO_6_ [7].

The *A* position in the rosiaite structure can be taken also by a trivalent rare-earth metal [8,9,10,11,12,13] or bismuth [14]. Simultaneously, in accord with charge compensation, half of *B* positions can be occupied by a trivalent cation (e.g., Cr^3+^ or Fe^3+^), and another half by Te^6+^ ions. Concerning magnetic properties, it is known that all RCrTeO_6_ studied (R = Y, La, Tb, Er, Gd, and Bi) experience the antiferromagnetic transition at low temperatures [11,12,14]. Paramagnetic behavior down to 3 K was observed in LaFeTeO_6_ [10] and GdFeTeO_6_ [13], albeit in the latter case it has been anticipated that this compound experiences ferrimagnetic order at lower temperature.

It is well known that in two dimensions, the triangular lattice antiferromagnet in the Ising limit remains disordered at all temperatures. In the Heisenberg limit, the problem of frustration is lifted by 120° arrangement of magnetic moments, which is the essence of Yafet–Kittel model [15]. The magnetic subsystem of GdFeTeO_6_ is comprised by basically isotropic Gd^3+^ (*J* = 7/2) and Fe^3+^ (*S* = 5/2) ions. It opens the way to unique clockwise/anticlockwise 120° antiferromagnetic structure in case of appreciable *f*–*d* interaction. The reported bond lengths in GdFeTeO_6_ [13] differ from the corresponding sums of ionic radii [16] by 0.16, 0.24 and 0.29 Å for Gd–O, Fe–O and Te–O, respectively. We report here the preparation, correctly refined crystal structure and detailed experimental and theoretical study of magnetic properties of GdFeTeO_6_ in comparison with its analogue containing diamagnetic Ga^3+^ in place of Fe^3+^.

## 2. Experimental

### 2.1. Sample Preparation, Phase Analysis and Structural Studies

Polycrystalline samples of GdFeTeO_6_ and GdGaTeO_6_ were prepared by solid-state reactions (see Electronic Supplementary Material, ESM, for details). Reasonable agreement of the hexagonal lattice parameters for GdFeTeO_6_ prepared by different methods, even in the presence of foreign phases (Table S1 of ESM), suggests that the compound does not have any extended homogeneity range.

XRD studies were performed in CuK_α_ radiation using an ARL X’tra diffractometer, Thermo Scientific, Switzerland, in the Bragg–Brentano geometry, equipped with a solid-state Si(Li) detector. Lattice parameters were refined using CELREF 3 (J. Laugier and B. Bochu), with angular corrections by corundum (NIST SRM 676) as an internal standard. To reduce effect of grain orientation, the samples for the XRD profile analysis were mixed with amorphous powder (instant coffee). The structures were refined with the GSAS + EXPGUI suite [17,18].

### 2.2. Physical Measurements

Magnetization *M* and specific heat *C*_p_ were studied using various options of Physical Properties Measurements System PPMS–9T, Quantum Design, San Diego, CA, USA.

## 3. Results and Discussion

### 3.1. Crystal Structures of GdMTeO_6_ (M = Fe, Ga)

For the structure analysis, we used a single-phase light yellow GdFeTeO_6_ powder prepared by solid-state reaction at a final temperature of 830 °C and white GdGaTeO_6_ powder prepared at 950 °C. The latter contained trace amounts of Ga_2_O_3_ and an unknown phase, and this may explain somewhat lower accuracy of its structural data (see below). The crystal structures were successfully refined starting from the structural model of LaFeTeO_6_ [10].

**Figure 1:**
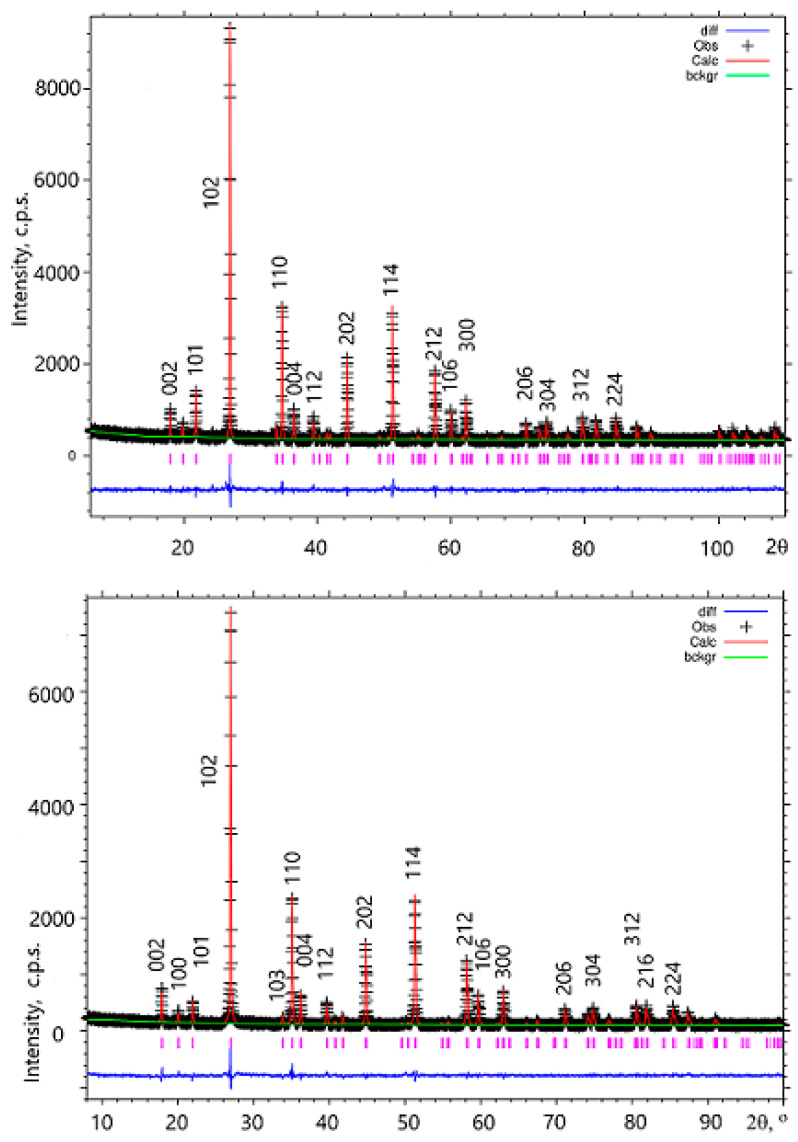
Results of the Rietveld refinement for GdFeTeO_6_ (**top**) and GdGaTeO_6_ (**bottom**). Crosses, experimental data; red line, calculated profile; bottom blue line, difference profile; vertical bars, Bragg angles.

Experimental and calculated XRD profiles are compared in Figure 1; experimental and refinement details, crystallographic data, atomic coordinates and displacement parameters are reported in Tables S2–S4 and Figure S2 of ESM, and bond lengths, bond angles and bond valence sums (BVS), in Table 1. The Crystallographic information files (CIF) are supplied as Supplementary Material. The data for GdFeTeO_6_ have been deposited at the Cambridge Crystallographic Data Centre as CCDC 2002460.

**Figure 2:**
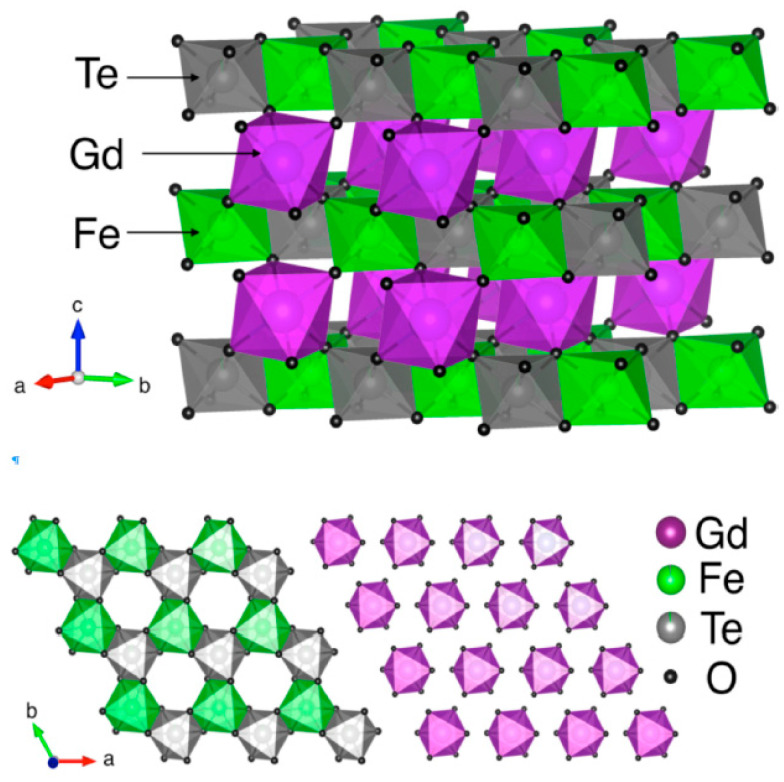
Upper panel: Polyhedral presentation of the crystal structure of GdFeTeO_6_, Violet, green, grey and black spheres are Gd, Fe, Te and O ions, respectively. Lower panel: magnetoactive layer of Fe and Te ions, and Gd layer. A three-dimensional visualization system VESTA [20] for electronic and structural analysis has been used for the presentation of crystal structure.

For the Fe compound, possible Te/Fe inversion was investigated, but the refined degree of inversion, 0.02, was within experimental accuracy and, therefore, was neglected. The structures are very similar and Figure 2 effectively represents both of them. Bond lengths agree with the corresponding sums of ionic radii (less accurately for M = Ga), and calculated BVS are also reasonable. Each oxygen anion has an almost planar environment, with the sum of the three bond angles being close to 360° (Table 1).

### 3.2. Basic Properties

**Figure 3:**
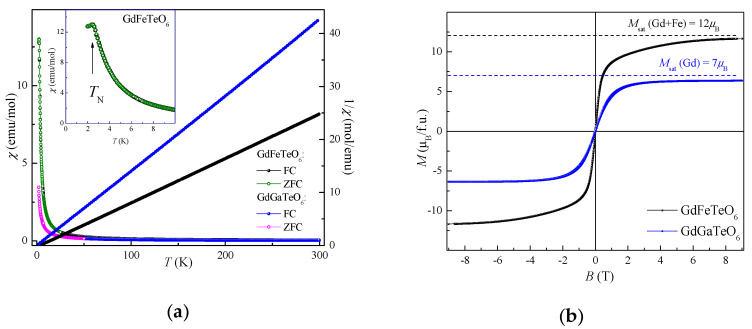
(**a**) The temperature dependences of magnetic susceptibility *χ* in GdFeTeO_6_ and GdGaTeO_6_ at *B* = 0.1 T (left ordinate) and inverse susceptibility 1/*χ* (right ordinate). The inset enlarges the low temperature part of *χ*(*T*) curve for GdFeTeO_6_. (**b**) The field dependences of magnetization *M*(*B*) in GdFeTeO_6_ and GdGaTeO_6_ at 2 K.

The temperature dependences of magnetic susceptibility *χ* = *M*/*B* in both GdFeTeO_6_ and GdGaTeO_6_ taken at *B* = 0.1 T are shown in the left panel of Figure 3. Note that no difference between data obtained within field-cooled and zero-field-cooled protocols has been observed signaling absence of any impurity-driven or spin-glass effects. In a wide temperature range, both compounds evidence paramagnetic behavior following basically the Curie law *χ* = *C*/*T*. The Curie constants *C* are 11.96 emu K/mol for GdFeTeO_6_ and 7.58 emu K/mol for GdGaTeO_6_ in full correspondence with expectations for Gd^3+^ (*J* = 7/2) and Fe^3+^ (*S* = 5/2) magnetic moments. Straight inverse susceptibility curves, *χ*^−1^(*T*), point to the absence of any magnetic frustration effects inherent to triangular systems. While GdGaTeO_6_ remains paramagnetic down to 2 K, the *χ*(*T*) curve in GdFeTeO_6_ evidences the kink ascribed to antiferromagnetic phase transition at *T*_N_ = 2.4 K. This part of the *χ*(*T*) curve is enlarged in the inset to the left panel of Figure 3. The kink at 2.4 K is readily suppressed by an external magnetic field. The field dependences of magnetization *M*(*B*) in GdFeTeO_6_ and GdGaTeO_6_ taken at 2 K are shown in the right panel of Figure 3. In both compounds, the magnetization reaches the saturation values, albeit the shape of *M*(*B*) curve in GdFeTeO_6_ differs from that in GdGaTeO_6_, presumably due to *f*–*d* interactions which result in magnetic long-range order.

**Figure 4:**
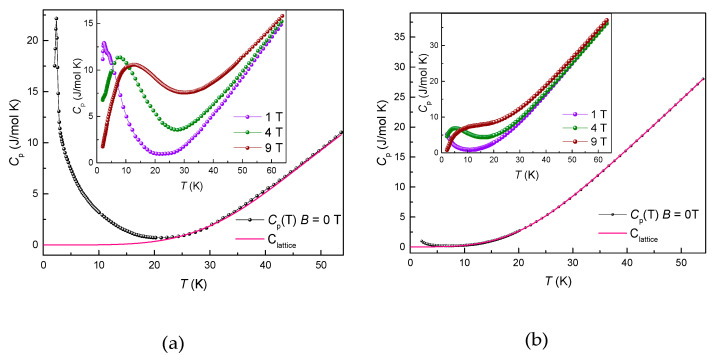
The temperature dependences of the specific heat *C*_p_ in GdFeTeO_6_ (**a**) and GdGaTeO_6_ (**b**). The solid lines represent the Debye fitting of the phonon contributions. The insets show *C*_p_(*T*) curves taken in various magnetic fields.

The evidence for the magnetic phase transition in GdFeTeO_6_ was further obtained from the specific heat data, as shown in the left panel of Figure 4. The *C*_p_(*T*) curve taken in the absence of magnetic field evidences sharp λ-type anomaly at *T*_N_ = 2.4 K. The data well above the transition temperature were used to estimate the phonon background *C*_lattice_ in GdFeTeO_6_ using the Debye model, as shown by the solid line in Figure 4. No such anomaly has been detected down to 2 K in GdGaTeO_6_; however, a slight upturn of specific heat at lowest temperatures should be noted which can be considered as an indication for the forthcoming low-temperature magnetic phase transition. An external magnetic field rapidly suppresses the λ-peak in *C*_p_(*T*) curve, but the Schottky-type anomaly appears at elevated temperatures. This anomaly is associated with the Zeeman splitting of magnetic levels in both gadolinium and iron ions. It shifts to higher temperatures with increasing magnetic field. Similarly, the Zeeman splitting of gadolinium levels in GdGaTeO_6_ results in the appearance of pronounced Schottky anomaly, as shown in the right panel of Figure 4.

### 3.3. Magnetocaloric Effect

**Figure 5:**
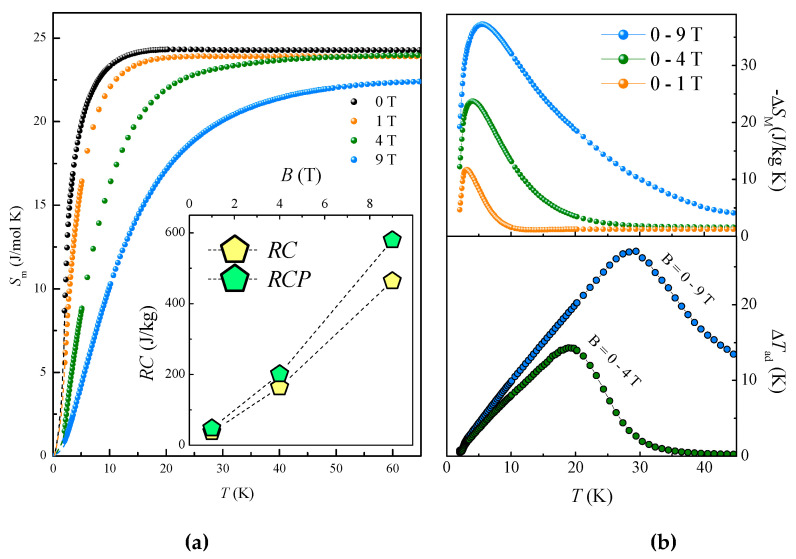
The temperature dependence of the magnetic entropy *S_M_* in various magnetic fields. The inset shows the field dependences of the refrigerant capacity *RC* and the relative cooling power *RCP* (**a**). The magnetic entropy Δ*S*_M_ and adiabatic temperature change Δ*T*_ad_ (**b**).

The magnetocaloric effect in GdFeTeO_6_ was estimated from the specific heat data *C*_p_(*T*, *B*) at various magnetic fields. The lattice contribution *C*_lattice_ has been subtracted from the total specific heat *C*_p_(*T*, *B*) to estimate the magnetic entropy *S*_m_(T), as shown in the left panel of Figure 5. The magnetic entropy change Δ*S*_M_(*T*, Δ*B*) has been calculated as Δ*S*_M_(*T*, Δ*B*) = *S*_m_(*T*, *B*) − S_m_(*T*, 0). The adiabatic change of the magnetic field from *B*_1_ to *B*_2_ causes not only change in the magnetic entropy, but also alteration of the sample temperature Δ*T*_ad_ = *T*_2_ − *T*_1_, which can be determined by the adiabatic condition *S*(*T*, *B*_1_) = *S*(*T* + Δ*T*, *B*_2_). Thus, Δ*T*_ad_ has been calculated from the zero-field *C*_p_(*T*) and Δ*S*_M_(*T*, Δ*B*) data with the use of equation:

(1) $$\Delta T\left(T,\Delta B\right)=T\left[\exp\left(-\frac{dS_{M}\left(T,\Delta B\right)}{C_{p}\left(T,0\right)}\right)-1\right]$$

Right panels of Figure 5 show both Δ*S*_M_ and Δ*T*_ad_ sets of data in GdFeTeO_6_ as a function of temperature for the various external magnetic fields. These curves demonstrate a broad peak for both quantities, with the width increasing at higher magnetic fields. The maximum value −Δ*S*_M_^max^ is 35.3 J/kg K for the field change of 0–9 T. This result agrees with the previous data for Δ*S*_M_(*T*, Δ*B*) derived from an isothermal process of magnetization using the Maxwell relations. The temperature change, Δ*T*_ad_, is 27 K at 9 T reaching liquid hydrogen temperatures.

The magnetocaloric study is completed by the estimation of refrigeration properties for GdFeTeO_6_. The results for the refrigerant capacity (RC) and the relative cooling power (RCP) vs. applied magnetic field are shown in the inset to the right panel of Figure 5. The RC and the RCP are defined as

(2) $$RC=\overset{T_{2}}{\underset{T_{1}}{\int }}\left|\Delta S_{M}dT\right|,RCP=\left|\Delta S_{M}^{max}\right|\times \left|\delta T_{FWHM}\right|$$

where *T*_1_ and *T*_2_ represent the temperatures corresponding to the half maximum of Δ*S_M_*(*T*) curve, Δ*S_M_^max^* is the maximum of Δ*S_M_* value, and δ*T_FWHM_* is the width at half maximum (FWHM) of Δ*S_M_*(*T*) curve. Note that similarly large magnetocaloric effect has been recently reported for another *f*-*d* oxide of rosiaite-type GdCrTeO_6_ [12].

### 3.4. Density Functional Calculations

Density functional theory calculations [21,22] were performed to study the magnetic interactions by determining the exchange interactions. We considered both Perdew–Burke–Ernzerhof (PBE + U) [23,24] and Heyd–Scuseria–Ernzerhof (HSE) [25,26] exchange correlation functionals. For the PBE + U calculations, the choice of *U* for the *f* electrons is subtle since different values have been deemed a reasonable choice for each specific material. For example, in elemental Gd [27] and Gd monopnictides [28], a *U_eff_* = *U*-*J* value of 6 eV has been used, but in some transition metal oxides a lower value of 4 eV has been found more appropriate for the *f* electrons of Gd and Dy [29,30]. In the previous study for this compound, a value of *U*_Gd_ = 6 eV has been used [13]. However, we found that the energy difference between different magnetic configurations is very sensitive to *U*_Gd_. Therefore, previous calculations for similar compounds must be considered with care with respect to the choice of the *U* parameter. In order to avoid ambiguities in the choice of *U* correction, we have used the HSE functional calculations as a benchmark. The energy differences are more sensitive to *U*_Gd_, with a value *U*_Gd_ close to 2.09 eV needed, but much less sensitive to *U*_Fe_, with 4 *U*_Fe_ = 5 eV producing similar energy differences. Nevertheless, there is no single combination of *U* values which can reproduce all the HSE energy differences between magnetic states, which prompted us to adopt the latter approach also in the estimate of exchange interactions. This is probably due to the fact that while HSE affects both *p*- and *d*-states, the *U* correction affects mainly the *d*-states [31].

We considered the 2 × 1 × 1 supercell in order to estimate the pairwise exchange interactions. Four Gd and four Fe atoms are considered, hence 9 possible AFM states, since both Fe and Gd can have three different AFM spin arrangements. We considered the FM, the nine AFM states and five additional FiM states, both for HSE and PBE + U, with *U*_Gd_ = 2.09, *U*_Fe_ = 5 eV, which was found to be a reasonable compromise to reproduce the HSE energies. We evaluated the exchange interactions by mapping the first-principles magnetic energies to the Heisenberg model:

(3) $$H=\frac{1}{2}\underset{i,j}{\sum }J_{i,j}{\vec{S}}_{i}\Delta {\vec{S}}_{j}$$

and performing multiple linear regression with least-squares fitting. Gd–Gd interactions were found to be negligible, and the magnetic energies can be well reproduced in a minimal model comprising only Fe–Fe and nearest-neighbor Gd-Fe interactions. The third nearest-neighbor out-of-plane Fe–Fe interaction was found to be important for improving the linear regression, even though the distance between involved Fe atoms is quite large; this exchange is in fact mediated by the same bridging atoms involved in the other Fe–Fe exchanges, occurring on a Fe–O–Gd–O–Fe path. The estimated values of the exchange interactions for PBE + U and HSE approaches are shown in Table 2. The interactions within HSE are generally reduced in comparison with PBE + U, with the main difference being a much smaller Fe–Fe in-plane interaction, and the sign of the next-nearest-neighbor $J_{Fe-Fe}^{out-of-plane}$, which turned out to be the only ferromagnetic interaction at the HSE or PBE + U levels. The in-plane AFM Fe–Fe interaction is known to lead to a (classical) co-planar three-sublattice “120-degree” state within each Fe-layer, while the Gd–Fe AFM interaction, mediating an inter-layer coupling between Fe layers, would instead promote a ferrimagnetic state with antiparallel Fe and Gd spins, and the third nearest-neighbor $J_{\mathrm{Fe}-\mathrm{Fe}}^{nnnout-of-plane}$ would instead favor AFM configurations of Fe spins. Such a fierce competition of magnetic exchange interactions combined with their weakness in magnitude is compatible with the low critical temperature observed in the system. Indeed, we used the estimated interactions to predict the critical temperature within a classical Monte Carlo approach. Using a Metropolis algorithm for an 18 × 18 × 12 cell, we found cusps in the specific heat and magnetic susceptibility signaling a transition at *T*_N_ ~ 2.6 K (5 K) for exchange parameters evaluated within the HSE (PBE + U) approach, with no net magnetization developing in the ordered phase, in good agreement with the experimental estimates.

The HSE (PBE + U) calculated local magnetic moments have absolute values of approximately 6.92, 4.25, 0.00, and 0.10 (6.97, 4.29, 0.01, and 0.09) *µ*_B_, for Gd, Fe, Te, and O, respectively, irrespective of the magnetic order considered, consistent with Gd^3+^ (*J* = 7/2) and Fe^3+^ (*S* = 5/2) ions, in agreement with the susceptibility measurements.

### 3.5. Discussion

The crystal structure of both GdFeTeO_6_ and GdGaTeO_6_ is a superstructure (space group $P\bar{3}1c$) of the rosiaite *AB_2_O_6_* [3] due to the doubling of the *c* parameter associated with the alternating Fe/Te or Ga/Te along the principal axis. Thermodynamic measurements evidence the long-range magnetic order at *T*_N_ = 2.4 K in GdFeTeO_6_ and paramagnetic behavior down to 2 K in GdGaTeO_6_. GdFeTeO_6_ possesses excellent magnetocaloric properties. For a magnetic field change of 9 T at 2 K, the values of entropy change −Δ*S*_M_(*T*) = 35.3 J/kg K and adiabatic temperature alteration Δ*T*_ad_ = 27 K are obtained, as well as relative cooling power 580 J/kg and refrigerant capacity 465 J/kg. This makes GdFeTeO_6_ attractive for the working body of low-temperature magnetic refrigerator devices. With the intensification of research at helium temperatures, the problem of cooling the current leads of superconducting solenoids and reaching ultralow temperatures becomes increasingly urgent [32,33,34,35,36,37].

**Figure 6:**
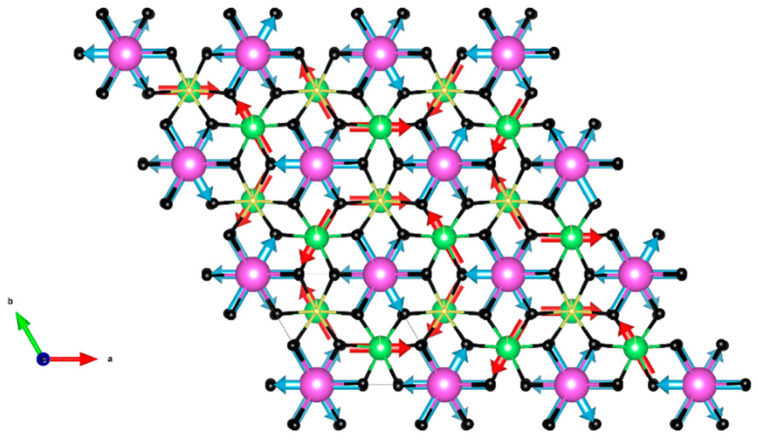
The ball-and-stick representation [20] of the crystal structure of GdFeTeO_6_. Purple, green, gray and black spheres are Gd, Fe, Te and O ions, respectively. Blue and pink arrows highlight the clockwise and anticlockwise arrangements of spins in different magnetoactive layers.

The presence of gadolinium in the structure of GdFeTeO_6_ precludes determination of its magnetic structure using neutron scattering methods. However, the absence of the magnetic order in GdGaTeO_6_ evidences the weakness of the Gd–Gd interactions. Moreover, no long-range order has been detected in LaFeTeO_6_, pointing to the weakness of interlayer Fe–Fe interactions [10]. GdFeTeO_6_ represents a unique system which orders due to interlayer f–d interactions. The absence of spontaneous magnetization in the magnetically ordered phase of this compound excludes any ferrimagnetic arrangement of Gd^3+^ and Fe^3+^ magnetic moments [13]. The only solution compatible with the absence of any frustration effects in magnetic susceptibility seems to be 120° structure of iron moments within B layers (say, clockwise) and 120° structure of gadolinium moments within A layers (say, anticlockwise), as shown in Figure 6. In the first principle calculations, this structure is of the lowest energy, but the energy difference with other configurations is very small, about 0.01 meV per formula unit. In our knowledge, the proposed chiral–antichiral magnetic structure is unique, deserving some kind of experimental verification.

## Figures and Tables

**Table 1:** Lattice parameters, bond lengths (Å), sums of ionic radii [16], bond valence [19] sums (BVS) and bond angles in GdMTeO_6_ (M = Fe, Ga).

		GdFeTeO_6_	GdGaTeO_6_
*a*, Å		5.16556(5)	5.11096(6)
*c*, Å		9.85231(14)	9.91922(17)
*c/a*		1.907	1.941
Distances/sum of radii/BVS	Gd-O	2.3283(10) × 6/2.30/2.73	2.280(10) × 6/2.30/3.06
	M-O	2.0141(9) × 6/2.005/3.01	1.929(10) × 6/1.98/3.43
	Te-O	1.9301(8) × 6/1.92/5.88	2.029(10) × 6/1.92/5.02
O: BVS		1.94	1.92
Angles (°)	Gd-O-Te	130.31(4)	126.8(5)
	Te-O-M	98.22(4)	96.4(4)
	M-O-Gd	125.78(4)	132.4(5)
	Sum for O	354.3	355.6
	O-Gd-O	88.0–92.0	86.2–93.8
	O-M-O	79.7–94.2	86.2–91.7
	O-Te-O	83.9–92.1	81.0–94.3

**Table 2:** Effective and renormalized (with *J*_Gd_ = 7/2, *S*_Fe_ = 5/2) exchange constants (meV) obtained by fitting to the PBE + U (*U*_Gd_ = 2.09, *U*_Fe_ = 5 eV) and HSE energies.

	Effective Exchange *J*_ij_^e^ = *S*_i_*S*_j_*J*_ij_ (meV)		*J*_ij_ for *J*_Gd_ = 7/2, *S*_Fe_ = 5/2 (meV)	
	PBE + U	HSE	PBE + U	HSE
$J_{Fe-Fe}^{in-plane}$	−0.4683	−0.0356	−0.0749	−0.0057
$J_{Fe-Fe}^{\mathrm{out}-\mathrm{of}-plane}$	−0.0263	0.0169	−0.0042	0.0027
$J_{Fe-Gd}$	−0.0382	−0.0993	−0.0044	−0.0113
$J_{\mathrm{Fe}-\mathrm{Fe}}^{nnnout-of-plane}$	−0.1831	−0.1182	−0.0293	−0.0189

## Data Availability

The data presented in this study are available in supplementary material.

## Supplementary Materials

The following are available online at https://www.mdpi.com/article/10.3390/ma14205954/s1, Figure S1. Comparison of the two XRD patterns of nominal “GdFeTeO_6_” taken in CuKα radia-tion. Upper panel: Relatively pure phase prepared from oxides for 48 h at 700 °C [5]. Lower pan-el: mixed phase prepared by the semi-wet method for 56 h at 750 °C with three intermediate re-grindings (this work), Figure S2. Correlation between crystal radii [6] of RE^3+^ and hexagonal subcell parameters of REFeTeO_6_. Red diamonds, a; blue triangles, c/2; RE = La [1, 3, 4], Pr, Nd, Sm [4], and Gd (this work); green circles, same from Reference [5], Figure S3. (a) Effect of changing UGd (eV) for the energy differences of one AFM and 3 FiM states with respect to the FM state. Changes of Gd UGd in the legend, with fixed UFe = 4 eV. (b) Energy difference between FiM1 and FM states. Effect of changing UGd and UFe, and comparison with HSE calculations (black line), Figure S4. The comparison of Monte-Carlo calculations (solid lines) with the experimental tem-perature dependencies of magnetic susceptibility χ(T) (left panel) and specific heat Cp(T) (right panel), Table S1. Phase analysis results and hexagonal lattice parameters of GdFeTeO_6_ samples prepared by various methods, Table S2. Details of the data collection and structure refinement, Table S3. Atomic coordinates and displacement parameters in GdFeTeO_6_, space group $P\bar{3}1c$, Table S4. Atomic coordinates and displacement parameters in GdGaTeO_6_, space group $P\bar{3}1c$.
