# Supplementary material for: Chirality and Magnetocaloricity in GdFeTeO6 as Compared to GdGaTeO6
Source: Materials (Basel). 2021 Oct 10;14(20):5954. doi: 10.3390/ma14205954 (PMC8539160; doi:10.3390/ma14205954)
Supplement: Supplementary file 1 [file materials-14-05954-s001.zip › materials-1399136-supplementary.pdf]

materials MDPI

Article

Elena Zvereva [1,#], Tatyana Vasilchikova [1], Maria Evstigneeva [2], Angelica Tyureva [2], Vladimir Nalbandyan [2], João Gonçalves [3], Paolo Barone [4], Alessandro Stroppa [5] and Alexander Vasiliev [1,6,*]

1 Department of Low Temperature Physics and Superconductivity, Physics Faculty, Lomonosov Moscow State University, 119991 Moscow, Russia; vasil@mig.phys.msu.ru (E.Z.); t_vasilchikova@yahoo.com (T.V.)
2 Department of Chemistry, Southern Federal University, 344090 Rostov-on-Don, Russia; maevstigneeva@gmail.com (M.E.); tyureva.lika@mail.ru (A.T.); vbn@sfedu.ru (V.N.)
3 CICECO, Aveiro University, 3810-193 Aveiro, Portugal; joaonsg@ua.pt
4 Consiglio Nazionale delle Ricerche, Institute for Superconducting and Innovative Materials and Devices (CNR-SPIN), Area della Ricerca di Tor Vergata, Via del Fosso del Cavaliere 100, 00133 Rome, Italy; paolo.barone@spin.cnr.it
5 Consiglio Nazionale delle Ricerche, Institute for Superconducting and Innovative Materials and Devices (CNR-SPIN), c/o Department of Physical and Chemical Sciences, University of L'Aquila, Via Vetoio I-67100 Coppito, L'Aquila, Italy; alessandro.stroppa@spin.cnr.it
6 Quantum Functional Materials Laboratory, National University of Science and Technology "MISiS", 119049 Moscow, Russia
Deceased
* Correspondence: anvas2000@yahoo.com

Citation: Zvereva, E.; Vasilchikova, T.; Evstigneeva, M.; Tyureva, A.; Nalbandyan, V.; Gonçalves, J.; Barone, P.; Stroppa, A.; Vasiliev, A. Chirality and Magnetocaloricity in $GdFeTeO_6$ as Compared to $GdGaTeO_6$. *Materials* **2021**, *14*, 5954. https://doi.org/10.3390/ma14205954

Academic Editor: Aivaras Kareiva

Received: 13 September 2021
Accepted: 29 September 2021
Published: 10 October 2021

Publisher's Note: MDPI stays neutral with regard to jurisdictional claims in published maps and institutional affiliations.

Copyright: © 2021 by the authors. Licensee MDPI, Basel, Switzerland. This article is an open access article distributed under the terms and conditions of the Creative Commons Attribution (CC BY) license (http://creativecommons.org/licenses/by/4.0/).

1. Starting Materials

All materials were of reagent grade. Due to the well-known problem of rare earth separation, admixtures of different rare earths in Gd_2O_3 might be suspected. To elucidate this point, energy dispersive X-ray (EDX) analysis was employed (INCA ENERGY 450/XT with an X-Act ADD detector). It has shown that our Gd_2O_3 is free from other rare earths or other metals within detection limits, far below 1 wt. %.

Hydrous ferric nitrate was analyzed gravimetrically after slow heating and calcination at 900 °C and then used in its pristine forms. However, its low stability in air and possible inhomogeneity resulted in poor accuracy of the composition. Therefore, for some syntheses we used an analyzed aqueous solution instead of the solid hydrate. Ferric oxide, gallium oxide, potassium carbonate and sodium vanadate were calcined at 400 °C, and gadolinium oxide, at 950 °C. Amorphous tellurium (VI) oxide could not be dried to constant weight, presumably due to continuous loss of oxygen. Therefore, it was used as received with 1–2% excess against the calculated amounts to compensate for possible adsorbed moisture and for possible loss during the high-temperature syntheses. K_2TeO_3 was prepared by reacting K_2CO_3 and TeO_3 for 3 h at 630 °C with intermediate regrinding and stored in a desiccator due to its strong hygroscopicity.

2. Preparation Methods

For the Fe compound, three preparation routes were tested:

(i) For the "semi-wet" route, a weighed portion of Gd_2O_3 was dissolved in aqueous nitric acid; a stoichiometric amount of ferric nitrate was added and the mixed solution was added dropwise to excess of aqueous ammonia solution under vigorous stirring. The coprecipitated hydroxide was aged, filtered, washed with distilled water under vacuum, and dried at 150 °C. Then, a stoichiometric portion of TeO_3 was admixed using mortar and pestle. The mixture was pressed and calcined, with intermittent regrinding and pressing, at 350–400 and 650–700 °C for 2 h each, and then at higher temperatures, as reported in Table S1.

(ii) For the conventional solid-state preparation, stoichiometric amounts of Gd_2O_3, TeO_3, and Fe_2O_3 were mixed by mortar and pestle and then processed as described above.

(iii) For the molten-salt method, 10 wt. % of either $NaVO_3$ or K_2TeO_3 were added to the starting or heat-treated mixture of reagents and then calcined at 700–800 °C followed by washing with water.

The Ga compound was prepared by the conventional solid-state method only, analogous to point (ii) above. The purest and well crystallized product was obtained after final heat treatment for 4 h at 950 °C.

Although tellurium oxide is known to be somewhat volatile [1,2] and toxic, the authors of the preceding works did not mention any precautions when preparing $AFeTeO_6$ for 24 h at 900 °C [3] or 2 h at 1000 °C [3]. TGA of $LaFeTeO_6$ [3] showed notable weight loss only above 1000 °C. However, this was done with the presynthesized compound under rapid heating. With prolonged heating of incompletely reacted mixtures, weight loss might start at lower temperatures. In the present work, all reactions at 800 °C and above were done in the fume hood using small covered Pt crucibles with reaction pellets packed within protective powder of the same composition but with added 0.5–2 wt. % excess TeO_3. Observed weight losses were very close to the excesses taken. Since other components are not volatile, this means that the Gd:Fe:Te ratio in the calcined pellet remained essentially the same as in the starting mixture, and the only problem was its phase composition (determined by XRD) rather than elemental composition. In any case, accuracy in weighing reagents is much better than that of the EDX method.

3. Phase Analysis of $GdFeTeO_6$

The reported preparation of well-crystallized $GdFeTeO_6$ from the raw oxides for 48 h at 700 °C [3] could not be reproduced not only by the solid-state method but even by the semi-wet method, despite heating at 750 °C for 56 h with three intermittent regrindings (Figure S1). On the other hand, molten-salt method, with $NaVO_3$ or K_2TeO_3, permitted preparation of the well-crystallized compound for shorter periods at 750–800 °C (Table S1). However, this brought admixtures of extra phases, e.g., $GdVO_4$, and might result in isomorphic substitutions, e.g., $Gd_{1-x}Na_xFe_{1-x}V_xTeO_6$.

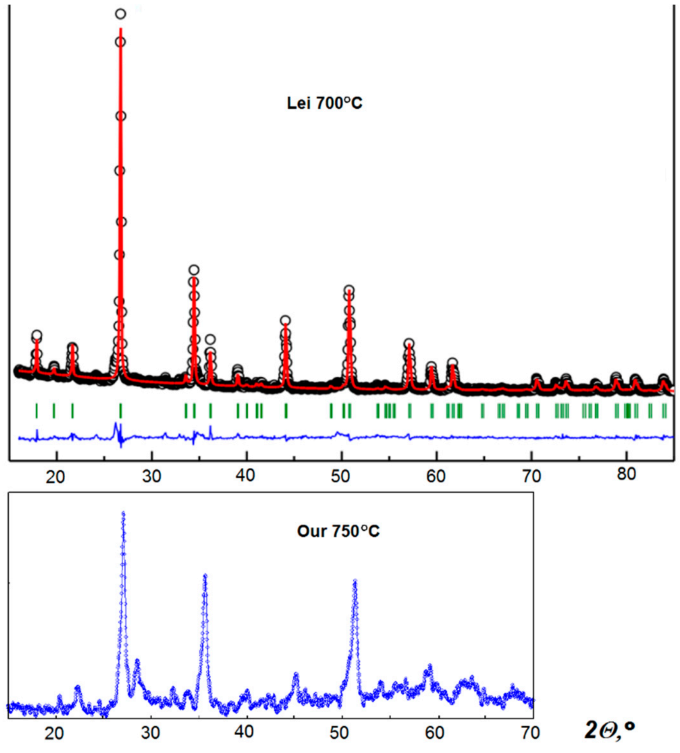

Figure S1. Comparison of the two XRD patterns of nominal "$GdFeTeO_6$" taken in CuKα radiation. Upper panel: Relatively pure phase prepared from oxides for 48 h at 700 °C [4]. Lower panel: mixed phase prepared by the semi-wet method for 56 h at 750 °C with three intermediate regrindings (this work).

Therefore, synthesis without extra components was necessary. It was accomplished at somewhat higher temperature of 830 °C. Results reported in Table S1 show that lattice parameters are essentially the same irrespective of the preparation method and foreign phases presence, thus making isomorphic admixtures less probable and suggesting that the phase does not have any extensive homogeneity range. It is also seen that too high temperature (e.g., 970 °C) did not enable preparation of the phase but the sample could be brought to essentially pure form by annealing at 830 °C. The compound is light yellow, turning orange with minor admixtures of Fe_2O_3 or Fe_2TeO_6.

Table S1. Phase analysis results and hexagonal lattice parameters of $GdFeTeO_6$ samples prepared by various methods.

#	Preparation method	Final heat treatment		*a*, Å	*c*, Å	Other phases
		t, °C	time, h			
1	Solid state	830	27	5.1662(2)	9.8529(1)	None
2	Semi-wet	830	27	5.163(3)	9.852(6)	Trace unknown
3	Semi-wet	970	3	5.1633(13)	9.8489(3)	Strong unknown, Fe_2O_3
4	Sample 3	830	27	5.1655(8)	9.854 (4)	Trace Fe_2TeO_6
5	Molten salt $NaVO_3$	750	20	5.1657(10)	9.8503(2)	$GdVO_4$, trace Fe_2TeO_6
6	Molten salt K_2TeO_3	800	5	5.1656(13)	9.8517(3)	Trace unknown

It should be noted that our lattice parameters for $GdFeTeO_6$ differ strongly from those reported previously for the same composition [4] but are in excellent correlation with those for four known analogues (Figure S2). This suggests that the powder pattern and lattice parameters reported by Lei et al. [4] might correspond to a different composition, because differences in Bragg angles of 0.5–0.8 Å and resulting differences in lattice parameters of 0.8% are far beyond possible experimental uncertainties.

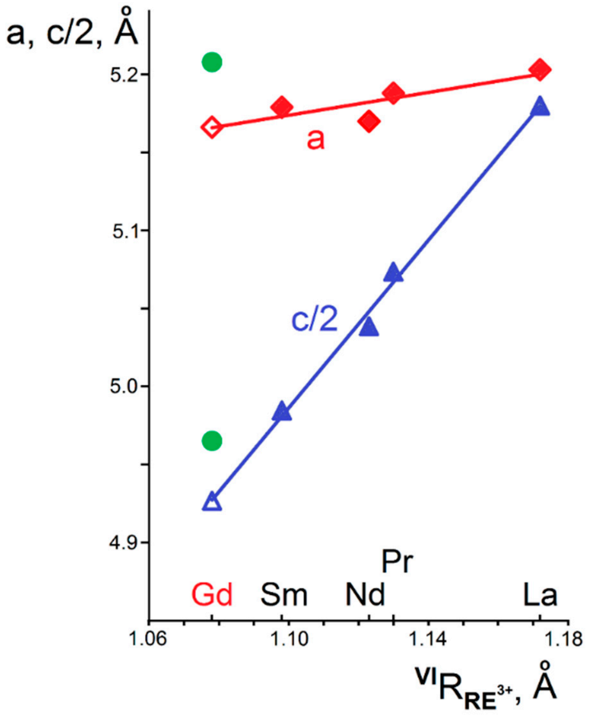

Figure S2. Correlation between crystal radii [5] of RE^{3+} and hexagonal subcell parameters of $REFeTeO_6$. Red diamonds, ***a***; blue triangles, ***c***/2; RE = La [1, 3, 6], Pr, Nd, Sm [6], and Gd (this work); green circles, same from Ref. [4].

4. Structural Data

Table S2. Details of the data collection and structure refinement.

		$GdFeTeO_6$	$GdGaTeO_6$
Crystal system		Trigonal	
Space group		$P\bar{3}1c$ (no. 163)	
Lattice constants	*a*, Å	5.16556(5)	5.11096(6)
	c, Å	9.85231(14)	9.91922(17)
Cell volume, $Å^3$		227.669(3)	224.395(4)
Formula weight		436.69	450.56
Z		2	2
Density (calc.), g/cm³		6.370	6.669
Wavelengths, Å	α_1	1.5406	
	α_2	1.5443	
	Ratio	0.5	
Polarization ratio		0.707	
Texture parameters (March-Dollase)		axis 001, ratio 1.00	
2Θ range, º		6.02–109.98	8.02–99.98
2Θ step, º		0.02	0.02
Anisotropic broadening		Axis 001	Axis 001
No. of data points		5199	4599
No. of reflections (α_1 only)		204	162
No. of variables		31	32
Agreement factors	$R(F^2)$	0.0952	0.0542
	R_p	0.0464	0.0745
	R_{wp}	0.0528	0.0974
	χ^2	1.50	1.68

Table S3. Atomic coordinates and displacement parameters in GdFeTeO6, space group $P\bar{3}1c$

Atom	Site	*x/a*	*y/b*	*z/c*	U_{iso}
Gd	2b	0	0	0	0.01297(7)
Fe	2c	1/3	2/3	¼	0.00206(11)
Te	2d	2/3	1/3	¼	0.01817(9)
O	12i	0.36772(20)	0.01242(17)	0.14103(7)	0.01371(11)

Table S4. Atomic coordinates and displacement parameters in $GdGaTeO_6$, space group $P\bar{3}1c$.

Atom	Site	*x/a*	*y/b*	*z/c*	U_{iso}
Gd	2b	0	0	0	0.01236
Ga	2c	1/3	2/3	1/4	0.00715
Te	2d	2/3	1/3	1/4	0.02455
O	12i	0.3442(25)	−0.0152(19)	0.1412(7)	0.01805

5. First-Principles Calculations: Technical Details and Functional Comparisons

We used the projector augmented-wave method [7] as implemented in VASP (version 5.4.4) [8]. Two types of exchange-correlation approximations were considered. First, the PBE+U approximation, with the generalized gradient approximation of Perdew, Burke and Ernzerhof (PBE) [9], and *U* as an additional effective on-site Coulomb interaction ap-

plied to strongly correlated Fe *d* and Gd *f* electrons, to improve their description [10]. Second, the Heyd-Scuseria-Ernzerhof (HSE) screened hybrid functional [11], where the short-range component of the PBE exchange is mixed with 25% Hartree-Fock exchange (we used the HSE06 version [12], with exchange screening parameter $\omega = 0.2$ Å^{-1}). The plane-waves energy cutoff of 400 eV, and a grid of 4 × 4 × 2 k-points in the unit cell were found to be enough for converged energy differences. For supercells similar k-point densities were used. Further convergence tests show that the computational set-up gives converged results. In our calculations we used the experimental structure for the atomic positions. The unit cell includes two Gd and two Fe atoms for a total of four magnetic moments.

We considered a total of five inequivalent collinear configurations that can be simulated with the same unit cell: a ferromagnetic (FM) configuration, an antiferromagnetic (AFM) one and three ferrimagnetic (FiM) states characterized by different relative orientations of Gd and Fe magnetic moments. Denoting each configuration as $M_{Gd}M_{Gd}M_{Fe}M_{Fe}$ where $M_X = u$ (d) represents up (down) spin for X = Gd, Fe atoms, the FiM1, FiM2, FiM3 configurations are defined as *uudd*, *uduu* and *uudu* respectively while the AFM and FM ones are defined as *udud* and *uuuu*, respectively. As shown in Fig. S3a, where we display the total energies for the different magnetic configurations with respect to the FM phase, the AFM, FiM1 and FiM3 states are almost degenerate within HSE, whereas the PBE+U approach tends to overestimate the antiferromagnetic interaction between Fe spins and, hence, to over-stabilize the AFM and FiM3 configurations with respect to HSE. The strong dependence of relative stability of the different magnetic configurations on U_{Gd} is highlighted in Fig. S3b, where we show the energy difference between FiM1 and FM configurations within PBE+U. Indeed, for $U_{Gd} \gtrsim 2.1$ eV the FM state appears to be more stable than the FiM1, while only values close to 2 eV can reasonably reproduce the HSE results. We also checked that U_{Fe} affects the energy differences much less, and values between 3 and 5 eV are reasonable choices when comparing to HSE results. However, we found that no combination of U_{Fe} and U_{Gd} can reproduce all the energy differences obtained by using HSE, which prompted us to adopt the latter approach also in the estimate of exchange interactions.

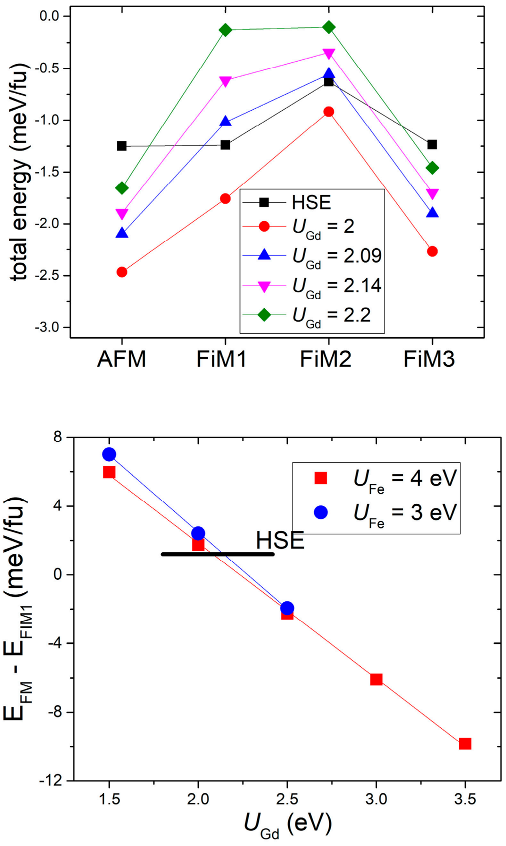

Figure S3. (a) Effect of changing U_{Gd} (eV) for the energy differences of one AFM and 3 FiM states with respect to the FM state. Changes of Gd U_{Gd} in the legend, with fixed U_{Fe} = 4 eV. (b) Energy difference between FiM1 and FM states. Effect of changing U_{Gd} and U_{Fe}, and comparison with HSE calculations (black line).

6. Monte Carlo Results: Specific Heat and Magnetic Susceptibility

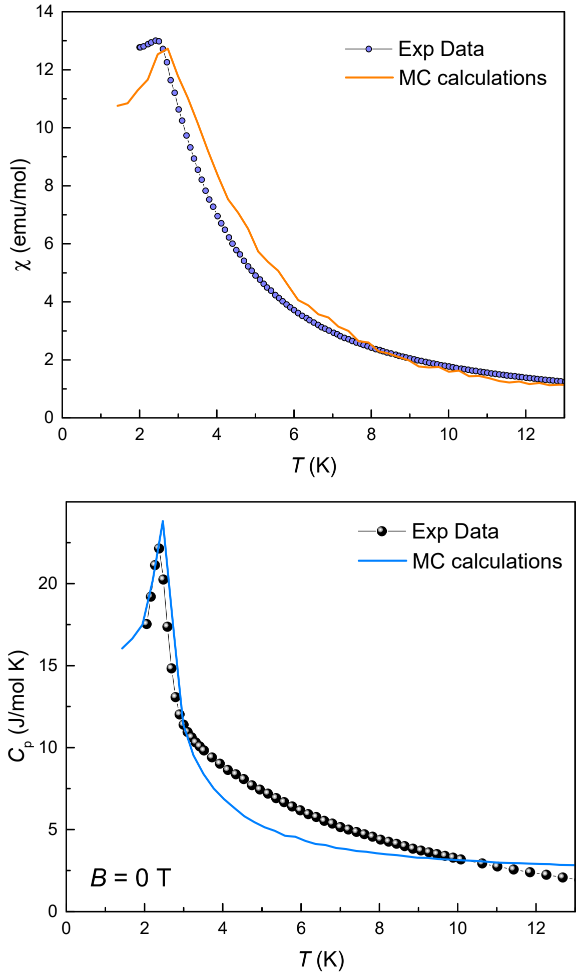

Figure S4. The comparison of Monte-Carlo calculations (solid lines) with the experimental temperature dependencies of magnetic susceptibility $\chi(T)$ (left panel) and specific heat $C_P(T)$ (right panel).

References

1. Gupta, N.; Roy, S.; Joshi, P.; Rao, G.R.; Krishnan, K.; Mudher, K.S. X-ray and thermal investigations of LaFeTeO6 and LaCrTeO6 compounds. *J. Alloys Compd*. **2006**, *417*, 300–303. DOI: 10.1016/j.jallcom.2005.09.044.
2. Roy, S.P.; Naik, Y.; Rao, G.A.R. Thermal investigations of SmFeTeO6 and SmCrTeO6 compounds. *J. Therm. Anal. Calorim.* **2013**, *114*, 1121–1124. DOI: 10.1007/s10973-013-3094-3.
3. Phatak, R.; Krishnan, K.; Kulkarni, N.; Achary, S.; Banerjee, A.; Sali, S. Crystal structure, magnetic and thermal properties of LaFeTeO6. *Mater. Res. Bull.* **2010**, *45*, 1978–1983. DOI: 10.1016/j.materresbull.2010.08.002
4. Lei, D.; Ouyang, Z.; Yue, X.; Yin, L.; Wang, Z.; Wang, J.; Xia, Z.; Rao, G. Weak magnetic interaction, large magnetocaloric effect, and underlying spin model in triangular lattice $GdFeTeO_6$. *J. Appl. Phys.* **2018**, *124*, 233904. DOI: 10.1063/1.5054675
5. Shannon, R.D. Revised effective ionic-radii and systematic studies of interatomic distances in halides and chalcogenides. *Acta Cryst. A* **1976**, *32*, 751–767. DOI: 10.1107/S0567739476001551
6. Lavat, A.; Mercader, C.; Baran, E. Crystallographic and spectroscopic characterization of LnFeTeO6 (Ln = La, Pr, Nd, Sm) materials. *J. Alloys Compd.* **2010**, *508*, 24–27. DOI: 10.1016/j.jallcom.2010.08.054
7. Blöchl, P. Projector augmented-wave method. *Phys. Rev. B* **1994**, *50*, 17953–17979. DOI: 10.1103/PhysRevB.50.17953
8. Kresse G.; Furthmüller, J. Efficient iterative schemes for ab initio total-energy calculations using a plane-wave basis set. *Phys. Rev. B* **1996**, *54*, 11169–11186. DOI: 10.1103/PhysRevB.54.11169
9. Perdew, J.; Burke, K.; Ernzerhof, M. Generalized Gradient Approximation Made Simple. *Phys. Rev. Lett.* **1996**, *77*, 3865–3868. DOI: 10.1103/PhysRevLett.77.3865
10. Dudarev, S.; Botton, G.; Savrasov, S.; Humphreys, C.; Sutton, A. Electron-energy-loss spectra and the structural stability of nickel oxide: An LSDA+U study. *Phys. Rev. B* **1998**, *57*, 1505–1509. DOI: 10.1103/PhysRevB.57.1505
11. Heyd, J.; Scuseria, G.; Ernzerhof, M. Hybrid functionals based on a screened Coulomb potential. *J. Chem. Phys.* **2003**, *118*, 8207-8215. DOI: 10.1063/1.1564060
12. Krukau, A.; Vydrov, O.; Izmaylov, A.; Scuseria, G. Influence of the exchange screening parameter on the performance of screened hybrid functionals. *J. Chem. Phys.* **2006**, *125*, 224106. DOI: 10.1063/1.2404663
